# Second Report of Accidental Intestinal Myiasis due to* Eristalis tenax* (Diptera: Syrphidae) in Iran, 2015

**DOI:** 10.1155/2017/3754180

**Published:** 2017-07-11

**Authors:** Ramezani Awal Riabi Hamed, Ramezani Awal Riabi Hamid, Naghizade Hamid

**Affiliations:** ^1^Faculty of Health, Department of Public Health, Gonabad University of Medical Sciences, Gonabad, Iran; ^2^Department of Basic Sciences, Gonabad University of Medical Sciences, Gonabad, Iran

## Abstract

We have described a new case of accidental intestinal myiasis that had occurred due to* Eristalis tenax* in Iran. A 4-year-old girl living in rural area near Bajestan city located in the south of Khorasan Razavi province visited the hospital lab with complaints of one live larva in feces and did not have other symptoms, except anal itching. This case had a history of consuming subterranean village water and did not have a history of traveling outside the city or contact with other patients.* Conclusion*. Based on the morphology characteristic, the larva was identified as “rat-tailed maggot” or larvae fly* E. tenax*.

## 1. Introduction

Myiasis is the infection of tissues or organs of vertebrate animals and humans by fly larvae. Myiasis frequently occurs in livestock and pets in rural areas. In humans, myiasis occurs primarily in unhealthy individuals in third-world countries [1]. Most infected organs are skin wounds, eyes, nose, nasal sinuses, throat, and intestines [2]. Human myiasis may be benign and asymptomatic or may lead to serious problems or even death.

Flies of the order Diptera are responsible for myiasis that belongs to genera* Sarcophaga* and* Calliphora*; among them, the Syrphidae family includes* Eristalis* genus, which has largely been dealt with in medicine [3].

Since the parasitic stage of its larvae is not obligate, the* Eristalis* genus is responsible for only a facultative kind of human myiasis. Within this genus, the fly* Eristalis tenax* has been the most described species. Despite the worldwide distribution of* E. tenax*, most cases of myiasis caused by the larvae of this species have occurred in developing countries where standards of hygiene are low [4].

In the present study, we describe the detection of* E. tenax* larva in feces, which is the second report of an infected human in Iran.

## 2. Case Presentation

We have described a new case of accidental intestinal myiasis that occurred due to* E. tenax* in Khorasan Razavi province of Iran. Only about 40 cases have been reported worldwide; two of them occurred in Iran. A 4-year-old girl living in a rural area near Bajestan city located in the south of Khorasan Razavi province visited the hospital lab with complaints of one live larva in feces and had no other symptoms except anal itching. The patient consumed subterranean village water and did not have a history of travel outside of the city and had not visited any outpatients. The patient returned home without treatment.

**Figure 1:**
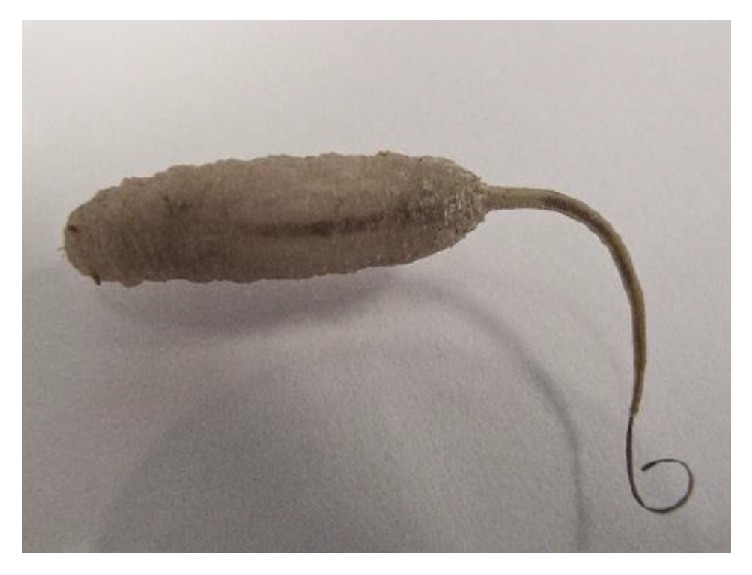
Larva of the rat-tailed maggot,* Eristalis tenax,* about 2.6 ± 0.7 cm in length.

The larvae sample was taken from the patient in the toilet, using plastic stools, which was immediately transferred to the Entomology Department Laboratory of Gonabad University of Medical Sciences. The specimen obtained from the patient was a cylindrical larva measuring about 2.6 ± 0.7 cm long with a tail. Hartley's identification key was used to identify the larva. Based on morphology characteristics, this larva has a cylindrical shape with patches of horizontal folds dividing the body into segments, between which the cuticle is smooth. At the segment of body, two rows of soft hairs are visible [5]. The larva has a siphon that acts as a respiratory attachment and also is like a tail, thus giving them their nickname “rat-tailed maggot.” The siphon can be several times the length of the body; it was identified as “rat-tailed maggot” or larvae fly* E. tenax* (Figure 1).

## 3. Discussion

*E. tenax* is the most common species of* Tubifera tenax* genera (Diptera: Syrphidae), and the posterior respiratory structure of their larvae gives them the name “rat-tailed larvae.” This appendix allows the aquatic larvae to breathe air while inhabiting highly polluted water. It constitutes three segments in which the apical segment holds a pair of spiracles. Moreover, the subapical segment can be drawn back into the basal segment [6].

Both adults and immature stages are potential vectors of mycobacterium [4, 7]. Water supplies and habits of patients are factors that should be considered in the evaluation of myiasis. Intestinal myiasis in humans perhaps raises concerns on accidental patterns of infection and follows consumption of contaminated raw or uncooked food or water. Herein, some ingested larvae are not destroyed by the digestive fluid, decay organic matter, and may produce various and unspecific gastrointestinal distress [8].

Analogous to our study, laboratories tests show that live larvae are predominantly instigated by intestinal myiasis flies. For instance, a 53-year-old French woman without relevant medical history was referred to the hospital laboratory for supposed release of larvae in her stools. Visiting the patient, physical examinations were apparently normal, until she excreted a larva while exerting gas from her rectal region. Analyzing the specimen revealed larva of the drone fly* E. tenax* species [4].

In November 2009, a 51-year-old Spanish woman, without relevant medical history, was referred to Royo Villanova Hospital in Zaragoza, Spain, due to presence of larvae in her feces. Macroscopic examination of the identified larva revealed a 1 cm body and a tail-like breathing tube with a distinctive terminal telescopic section measuring 2 cm, which was identified as* E. tenax* [9]. During summer of 2003, a 36-year-old Belgian man visited his physician with the chief complaint of diarrhea and intestinal grumbling. Larvae released on numerous instances were recognized as* E. tenax* [10].

Since human infestations due to* E*.* tenax *larvae have so far probably been neglected, only 40 cases have been reported [11].

In the 40 reported cases the degree and presence of symptoms, mostly nonspecific, likely depended on the parasite burden ranging from diarrhea and abdominal pains to proctitis or anal pruritus. Demographics of patients were unlike and 52% of cases were women. The average age of patients was 36 years including infants to the elderly (1–64 years). The distribution was widespread from tropical areas to semiarid regions and zones with temperate climates. Most cases have been reported in developing countries where sanitary conditions are poor especially in rural environments with the exception of a handful of cases in urban areas.

The first intestinal myiasis of Iran (2010) was in a 22-year-old woman living in a rural area of Babol, Mazandaran [north of Iran], representing a history of excreting active swimming “fish-like creatures” in her stool. However, she had not experienced symptoms such as anal purities. Entomologists were identified such as “rat-tailed maggots” or larvae of* E. tenax* [12]. The first reported nasal myiasis case of* E. tenax* larva was in Iran [13]. The present study is the second report of myiasis from Iran. This occurrence was after five years of the first report, and similarly the patient was living in rural areas, and her hygienic conditions were inadequately poor.

## 4. Conclusion

According to the findings of this research, in tropical regions where there is a risk of infection of people in rural areas, clinicians and laboratory scientists should acquire complete understanding of fly larvae myiasis and offer appropriate treatment to visiting patients.
